# Anesthetic Management of a Super Morbidly Obese Obstetric Patient With a Body Mass Index of 109 kg/m2 Presenting for Her Fourth Caesarean Delivery

**DOI:** 10.7759/cureus.11803

**Published:** 2020-11-30

**Authors:** Derek K HO, Daniela S Karagyozyan, Taysir W Awad, Rashmi Vandse

**Affiliations:** 1 Anesthesiology, Loma Linda University Medical Center, Loma Linda, USA; 2 Anesthesiology, Emory University School of Medicine, Loma Linda, USA

**Keywords:** super super morbid obesity, difficult airway management, difficult epidural, postpartum haemorhage, repeat caesarean section

## Abstract

Morbidly obese obstetric patients undergoing anesthesia present many unique challenges. Previous caesarean sections (CSs) further complicate their management. We present the successful anesthetic management of a super morbidly obese obstetric patient with body mass index (BMI) of 109 kg/m^2^ who underwent her fourth CS. As per our review, this patient has the highest recorded BMI in the obstetric anesthesia literature.

A 27-year-old female, G4P3003, presented for fourth repeat CS at 38 weeks’ gestation. She had obstructive sleep apnea, hypertension, atrial fibrillation, and type 2 diabetes. Her first CS was emergent under general anesthesia (GA), and the other two were performed under neuraxial anesthesia, with the most recent one complicated by intraoperative cardiac arrest requiring cardiopulmonary resuscitation. Preoperative preparation involved multidisciplinary preparation, planning, and risk stratification. Although neuraxial anesthesia is preferred over GA for CS, she refused neuraxial anesthesia due to her prior traumatic experience and the potential that it caused her prior cardiac arrest. In addition, her inability to position for a block or lay flat, poor anatomical landmarks, unknown length of surgery, plan for periumbilical incision, uncertain placental status, and risk of massive hemorrhage convinced us to consider GA. Surprisingly, her airway examination was reassuring. Two 18G peripheral intravenous lines and an arterial line were obtained prior to induction. With optimum patient positioning and preoxygenation, modified rapid sequence induction with mask ventilation and endotracheal intubation with direct laryngoscopy were performed. A healthy baby was delivered without significant intraoperative complications. Intraoperative lung-protective strategy with recruitment maneuvers, multimodal analgesia, and elective postoperative continuous positive airway pressure aided in successful extubation. Postoperatively, pulmonary toilet, early mobilization, physical therapy, and venous thromboembolism prophylaxis were employed. Her postoperative course was complicated by severe preeclampsia and pulmonary embolism, which were managed successfully in the intensive care unit. She was discharged initially to outpatient rehabilitation followed by home. This case highlights the complexities and significance of an individualized approach in managing super morbidly obese obstetric patients.

## Introduction

Morbidly obese obstetric patients undergoing anesthesia present many unique challenges. These patients are at increased risk of maternal and perinatal complications [1-2]. There seems to be a “dose-response” relationship between the severity of maternal obesity and perinatal complications. Super obese parturients are at even higher risk compared to other morbidly obese parturients [3-5]. Multiple prior caesarean sections (CSs) further complicate their management [6]. Although neuraxial anesthesia is often preferred for elective CS, general anesthesia (GA) may be warranted in select patients. As per our literature review, our patient is unique as she is the first reported obstetric patient with a body mass index (BMI) of 109 kg/m^2^, who successfully underwent her fourth caesarean delivery under GA.

## Case presentation

Our patient was a 27-year-old African American female, G4P3003, with an intrauterine pregnancy at 38 weeks 2 days, who presented for an elective fourth repeat CS. She was morbidly obese, weighed 316 kilograms, and was 170 cm tall (BMI: 109 kg/m^2^). Her additional medical history included obstructive sleep apnea (OSA) noncompliant with continuous positive airway pressure (CPAP), hypertension, paroxysmal atrial fibrillation noncompliant with anticoagulation, type 2 diabetes mellitus, and gastroesophageal reflux disease. Her medications included prenatal vitamins and metoprolol.

Her first CS for fetal intolerance to labor was performed under GA followed by two repeat CSs with neuraxial blocks. Her most recent CS was complicated by intraoperative cardiac arrest requiring eight minutes of cardiopulmonary resuscitation prior to return of spontaneous circulation. The presumed cause was pulmonary embolism (PE); however, this was following a successful neuraxial block, and therefore the cause may have been hypotension that was not immediately recognized because of the patient habitus. Following this complication, the patient required a week-long intensive care unit (ICU) stay leading to significant deconditioning on top of her already morbidly obese state. Upon discharge, the patient was mostly bedbound, which led to a weight gain of over 250 pounds. Her prenatal care consisted solely of a single obstetric visit with ultrasound at 12 weeks. Social history was positive for marijuana use during pregnancy.

When she presented initially to our institution at a gestational age of 38 weeks 2 days, she complained of worsening shortness of breath. Given her prior history of possible PE, limited mobility, and atrial fibrillation without anticoagulation, suspicion for repeat PE was very high. However, she could not undergo computed tomography pulmonary angiography (CTPE) study, as a 500-pound weight limit precluded her from fitting in the scanner. Her transthoracic echocardiogram, which was also challenging, showed an ejection fraction of 70% with moderate left ventricular hypertrophy without any evidence of right heart strain. The patient’s shortness of breath subsequently improved, and the decision was made to perform a repeat CS with bilateral tubal ligation. Her basic metabolic panel and complete blood count were unremarkable.

The obstetric anesthesia team was consulted early along with other relevant subspecialties for multidisciplinary preparation, planning, and risk stratification. She was extremely nervous and refused neuraxial anesthesia due to her prior traumatic experience. Surprisingly, her airway examination was reassuring with Mallampati grade II with good neck extension, adequate mouth opening, and intact dentition. Adequate intravenous access, including two 18G peripheral intravenous lines, was obtained prior to induction. In addition, preparations for massive transfusion were made, including arterial access for monitoring as well as expected frequent blood sampling in case of hemorrhage. She was positioned carefully with adequate ramping to facilitate intubation. The difficult intubation cart was kept ready. She was preoxygenated for several minutes, but her end-tidal oxygen could not be brought to above 70% due to her body habitus. However, given her reassuring airway exam, we decided to induce. We used propofol 500 mg and succinylcholine 350 mg in a modified rapid sequence induction, with bag mask ventilation prior to intubation in order to bring up her oxygen reserve. We were able to achieve tidal volumes of 500 mL with an oral airway and two-hand ventilation. The first attempt with video laryngoscopy showed a large tongue and lots of redundant tissue leading to a brief desaturation to 80% and unsuccessful intubation. She was ventilated again using bag mask and subsequently intubated successfully by direct laryngoscopy, which was moderately difficult. She was ventilated with a low tidal volume strategy (6-8 mL/kg) based on her ideal body weight along with intermittent recruitment breaths to prevent atelectasis. We used an oxygen, air, and desflurane mixture as the inhaled anesthetic with FiO_^2^_ of 60%. Towards the end of the case, 50% nitrous oxide was used along with oxygen and desflurane. The dexmedetomidine infusion at 0.2mcg/kg/hour was administered throughout the case. The adequate depth of anesthesia was ensured by maintaining minimum alveolar concentration (MAC) of around 1 MAC. She also received famotidine 20 mg, metoclopramide 10 mg, dexamethasone 4 mg, and ondansetron 4 mg immediately after induction for prophylaxis against postoperative nausea. Rocuronium (total 50 mg) was administered in divided doses to maintain muscle relaxation.

The obstetricians performed a midline vertical supra-umbilical incision through a previous existing scar for improved surgical access to the uterus. They successfully delivered a 3,900-gram female neonate with Apgar scores of 4 and 7 at 1 and 5 minutes, respectively. The placenta separated immediately. The infusion of oxytocin 40 units/hour along with carboprost 250 mcg was administered to achieve adequate uterine tone. The total duration of surgery was 2 hours and 30 minutes. The estimated blood loss was approximately 1,500 mL; however, she remained hemodynamically stable. Multimodal pain control was employed using a combination of intravenous medications, including fentanyl (150 mcg) after the delivery, acetaminophen (1,000 mg), ketorolac (30 mg), and dexmedetomidine infusion (0.2 mcg/kg/hour). Neuromuscular blockade was monitored by peripheral nerve stimulator. Sugammadex (2 mg/kg actual body weight) was administered at the conclusion of the surgery as she already had four twitches to train-of-four (TOF) stimulation. She was successfully extubated and transitioned to oxygen delivery through a face mask.

She was electively placed on CPAP in the postanesthesia care unit (PACU). Her postoperative hemoglobin decreased significantly from 10.3 to 7.2 g/dL, requiring blood transfusion in the PACU. Postoperatively, care was taken to provide active physical therapy, venous thromboembolism (VTE) prophylaxis, multimodal pain management, and pulmonary hygiene, including incentive spirometry, chest physiotherapy, and positive expiratory pressure therapy to facilitate her recovery. However, her postoperative course was complicated by the development of preeclampsia with severe features. In addition, she developed acute shortness of breath and hypercarbic respiratory failure on postoperative day 3, requiring transfer to the surgical ICU for noninvasive positive pressure ventilation. She was started on anticoagulation for presumed PE along with treatment for preeclampsia. Her respiratory status improved over the next two days, repeat echocardiogram was unchanged from prior, and she was downgraded from the surgical ICU after two days and discharged from the hospital three days later to acute rehabilitation followed by home.

## Discussion

Obesity is a multisystem disease with several accompanying comorbidities including OSA, hypertension, preeclampsia, and gestational diabetes [1-2]. Obesity in pregnancy is an independent risk factor for maternal and neonatal complications such as hypoxemia, intrapartum fetal distress, failure to progress, instrumental delivery, and CS including anesthesia-related maternal mortality [1-2], whereas super morbid obesity further accentuates these risks [3-5]. The provision of anesthesia to an obese parturient is technically challenging due to difficulties associated with vascular access, patient positioning, monitoring, airway management, and performance of neuraxial anesthesia.

A patient with BMI > 40 kg/m^2^ is considered as morbidly obese, whereas a patient with BMI > 50 kg/m^2^ is classified as super morbidly obese [1-2]. The literature depicting the anesthetic management of obstetric patients with extreme morbid obesity is lacking. A review of existing literature yielded a case series involving three patients with extreme morbid obesity with BMI ranging from 73 to 95 kg/m^2^ [7] and two other case reports describing patients with BMI of 73 kg/m^2^ and 76 kg/m^2^ who underwent CS under neuraxial anesthesia [8-9]. Another complicating factor in our patient was her prior three CSs. Maternal morbidity rises in a dose-response manner with each additional CS, specifically in women with over three CSs who have increased risk of placenta previa, accreta, and hysterectomy [6]. Given the patient’s body habitus, obstetric ultrasound was not able to rule out the placental anomalies.

The multidisciplinary planning was initiated immediately. The obstetric anesthesia service was consulted early on for preoperative risk stratification and optimization. She was extremely anxious and required a lot of counselling and reassurance. Records from her previous hospitalization for repeat cesarean delivery complicated by cardiac arrest and subsequent ICU stay were obtained. We discussed with her the risks and benefits of neuraxial and GA while acknowledging the fact that either would be challenging in her case. We also disclosed her increased risk of maternal and fetal complications and the need for close perioperative monitoring.

The first decision to make was between neuraxial and GA. GA is not the preferred technique in obese patients due to higher incidence of difficult airway among obese parturients [1,10] along with the increased risk of aspiration, postpartum hemorrhage, and fetal exposure to volatile and intravenous anesthetics [2,8,10]. Our patient, however, refused neuraxial anesthesia due to her painful experience from her last CS. Nevertheless, her inability to lay flat or position for a block, difficulty in breathing at baseline, poor anatomical landmarks, unknown length of surgery, plan for periumbilical incision, uncertain placental status, and apparent risk of significant hemorrhage convinced us to choose GA over neuraxial anesthesia. Nonetheless, if one decides to use neuraxial anesthesia in obese parturients, the use of ultrasound for landmark identification has been shown to reduce procedure time, improve likelihood of first-pass success, and improve patient satisfaction [11-12].

We discussed the possibility of an awake fiberoptic intubation, as the physiological and anatomical changes produced by both obesity and pregnancy increased her potential for an unanticipated difficult airway. However, our patient became extremely anxious, and given her reassuring airway examination, we decided to proceed with an asleep video laryngoscopy for intubation in a ramped position with multiple antiemetics administered to decrease the risk of aspiration during induction. Our decision to use video laryngoscopy was based on the robust recommendation by the Obstetric Anesthetists’ Association and the Difficult Airway Society for using it for all intubations in obese parturients [1]. Even in a sitting position, she could not preoxygenate adequately. Given her increased risk of rapid desaturation during the apneic period secondary to decreased functional residual capacity and increased oxygen consumption, we decided to bag mask ventilate her after induction instead of a true rapid sequence induction. The use of nasal cannula insufflating oxygen at 5 L/min during intubation has been suggested to prolong the time to desaturation [2]. We consulted the American Society of Anesthesiologists’ difficult airway algorithm for potential backup plans if our asleep intubation were to fail, and we had backup supplies in the room, including oropharyngeal and nasopharyngeal airways, gum elastic bougies, supraglottic devices, and fiberoptic scopes along with extra personnel [13].

Her prior three CSs and super morbid obesity placed her at high risk of intraoperative and postpartum hemorrhage. The risk of accreta is 3.3% to 4% in patients with three prior CSs [6]. In addition, her body habitus increased her risk of hypotension both from postpartum hemorrhage and decreased preload due to increased compression of the inferior vena cava from her gravid uterus and abdominal weight [10]. We therefore performed left uterine displacement, placed a pre-induction arterial line along with adequate intravascular access, and made preparations for massive transfusion. The case was complicated by severe omental adhesions, an umbilical hernia, and postpartum hemorrhage requiring both oxytocin and carboprost to increase uterine tone and decrease uterine bleeding. We also had methylergonovine available for administration in the room. Blood loss was estimated to be 1,500 mL; however, she luckily did not have placenta accreta.

Super morbid obesity accentuates the physiological changes associated with pregnancy resulting in alterations in the pharmacokinetic properties of most of the anesthetic drugs [2]. The majority of the anesthetic drugs have not been studied in this patient population (BMI > 100 kg/m^2^). In general, the recommendation is to set the induction dose of propofol based on lean body weight (LBW), whereas the maintenance dose should be based on total body weight (TBW). Opioids such as fentanyl and remifentanil are also dosed based on LBW, non-depolarizing relaxants are dosed based on ideal body weight, and succinylcholine is dosed based on the TBW [2,14].

Managing intraoperative ventilation and preparing the patient for safe extubation required specific planning. We applied low tidal volume ventilation based on her ideal body weight along with periodic recruitment maneuvers and positive end-expiratory pressure, which have been shown to reduce atelectasis and improve oxygenation and lung compliance in obese patients [15-17]. This open lung concept is also implicated in preventing the development of ventilator-induced lung injury by stabilizing the alveoli. Extubation is a very critical period, and deaths have been reported due to hypoventilation and airway obstruction [1]. We kept the patient 45 degrees head up to improve the respiratory dynamics. Complete reversal of neuromuscular blockade was ensured by using sugammadex, although there is not much literature on its use in this patient group. CPAP was utilized in PACU to minimize the risks for hypoventilation, atelectasis, and hypoxemia.

Perioperative pain control was challenging due to the patient’s morbid obesity, OSA, chronic respiratory disease, deconditioning, and recent surgery. Pain control solely with opioids would have been more dangerous for the patient due to the risk of respiratory depression in combination with her comorbidities. We opted for a multimodal approach with low dose fentanyl, acetaminophen, and a dexmedetomidine infusion throughout the case. Multimodal analgesia continued in the PACU with non-opioid adjuvants including acetaminophen, gabapentin, ketorolac, and methocarbamol. Once the patient was more awake, hydromorphone was delivered through patient-controlled analgesia. Unfortunately, we were unable to utilize intrathecal or epidural long-acting opioids, which would have helped with pain control with minimal respiratory depression compared to intravenous opioids [2]. However, regional analgesia using transversus abdominis plane block could have been an option for her and has been shown to reduce opioid use when used as an adjunct for pain control after CSs [18].

Obese obstetric patients are at increased risk of postoperative complications, including cardiomyopathy, congestive heart failure, PE, and death [1,2,19]. Therefore, our patient was counseled rigorously to encourage her to participate in multiple measures in order to improve postoperative outcomes. These included early mobilization to a chair and strict participation with physical therapy and respiratory therapy. In addition, early VTE prophylaxis was started within 12 hours postoperatively followed by discharge to a rehabilitation facility. Some authors advocate for postoperative admission to the surgical ICU for closer postoperative monitoring [7]. Our patient ended up transferring to the surgical ICU following a presumed PE; therefore, this option would have been reasonable. Initial postoperative admission directly to the surgical ICU may alleviate some of these concerns with higher acuity of care.

## Conclusions

Our case illustrates the unique challenges that befall the anesthesia team for patients with extremely high BMI. It is essential to maintain good communication among all team members, carefully plan the entire perioperative course, and anticipate and prepare for any adverse events. The individualized approach that best applies to a particular patient should be implemented.
